# The iPRISM webtool: an interactive tool to pragmatically guide the iterative use of the Practical, Robust Implementation and Sustainability Model in public health and clinical settings

**DOI:** 10.1186/s43058-023-00494-4

**Published:** 2023-09-19

**Authors:** Katy E. Trinkley, Russell E. Glasgow, Sidney D’Mello, Meredith P. Fort, Bryan Ford, Borsika A. Rabin

**Affiliations:** 1 Department of Family Medicine, School of Medicine, University of Colorado Anschutz Medical Campus, 12631 E. 17th Ave., Mail Stop F496, Aurora, CO 80045 USA; 2 Adult and Child Center for Outcomes Research and Delivery Science Center, University of Colorado Anschutz Medical Campus, Aurora, CO USA; 3 Institute of Cognitive Science, University of Colorado Boulder, Boulder, CO USA; 4 Colorado School of Public Health, University of Colorado Anschutz Medical Campus, Aurora, CO USA; 5 Herbert Wertheim School of Public Health and Human Longevity Science, University of California San Diego, La Jolla, CA USA; 6 ACTRI Dissemination and Implementation Science Center, University of California San Diego, La Jolla, CA USA

**Keywords:** Implementation, Adaptation, PRISM, Translational science, Software tool, Program sustainability, Rapid research, Iterative

## Abstract

**Background:**

To increase uptake of implementation science (IS) methods by researchers and implementers, many have called for ways to make it more accessible and intuitive. The purpose of this paper is to describe the iPRISM webtool (Iterative, Practical, Robust Implementation and Sustainability Model) and how this interactive tool operationalizes PRISM to assess and guide a program’s (a) alignment with context, (b) progress on pragmatic outcomes, (c) potential adaptations, and (d) future sustainability across the stages of the implementation lifecycle.

**Methods:**

We used an iterative human-centered design process to develop the iPRISM webtool.

**Results:**

We conducted user-testing with 28 potential individual and team-based users who were English and Spanish speaking from diverse settings in various stages of implementing different types of programs. Users provided input on all aspects of the webtool including its purpose, content, assessment items, visual feedback displays, navigation, and potential application. Participants generally expressed interest in using the webtool and high likelihood of recommending it to others. The iPRISM webtool guides English and Spanish-speaking users through the process of iteratively applying PRISM across the lifecycle of a program to facilitate systematic assessment and alignment with context. The webtool summarizes assessment responses in graphical and tabular displays and then guides users to develop feasible and impactful adaptations and corresponding action plans. Equity considerations are integrated throughout.

**Conclusions:**

The iPRISM webtool can intuitively guide individuals and teams from diverse settings through the process of using IS methods to iteratively assess and adapt different types of programs to align with the context across the implementation lifecycle. Future research and application will continue to develop and evaluate this IS resource.

**Supplementary Information:**

The online version contains supplementary material available at 10.1186/s43058-023-00494-4.

Contributions to the literature

- The iPRISM webtool simplifies application of the Practical, Robust Implementation and Sustainability Model (PRISM) which includes the RE-AIM outcome measures.
- The iPRISM webtool can be used at any stage of an evidence-based program’s lifecycle to assess and align a program with the context, assess progress on outcomes of importance, inform the development of needed adaptations, and plan for sustainability.
- The iPRISM webtool can be used iteratively to guide adaptations.

## Introduction

Implementation science (IS) facilitates and studies the translation of relevant, sustainable, and reproducible evidence-based programs (EBP) into routine settings [1–5]. IS theories, models, and frameworks (TMFs) and methods aim to enhance the value, relevance, and impact of research and improve the translation of evidence to practice [6, 7]. A key predictor of an EBP’s uptake, implementation, and sustainment in real-world settings is fit or alignment with the context [3, 8, 9]. Lack of fit makes research less relevant and is an oversight of much research that has resulted in what many refer to as the “leaky pipeline” in which it takes 17 years for only 14% of evidence to ever make it into routine practice settings [2, 10]. IS models and methods aim to improve alignment between an EBP and context in real-world settings in a manner that can be reproduced in other real-world settings, helping to address the “failure to replicate crisis” [11]. Some evidence suggests that by applying IS models and methods, the lag in translating evidence to practice can be decreased to as little as 3–5 years and the amount of research integration can be increased as high as 80% [12, 13], which can enable rapid learning health systems [14].

Despite its promise and potential benefits, a key reason for the lack of IS uptake by the broad community of researchers and implementers lies in its complexity which can make it difficult to grasp for newcomers [15, 16]. For example, the growing numbers of TMFs and issues related to inconsistent and niche jargon make it difficult for the broad community of researchers and implementers to understand, synthesize, or apply TMF concepts to elevate the impact of their research [7, 17–22]. Furthermore, TMFs generally offer limited guidance on how to operationalize or make them actionable. Many of the TMFs and methods have also become overly complex or perceived as inflexible, lacking the pragmatic characteristics that make their application feasible or practical across diverse types of EBPs [15]. As it grew, the field of IS—which prioritizes real-world relevance—may have unintentionally made its methods difficult for the broad community of researchers and implementers to understand or apply [15].

To improve the uptake of IS TMFs and methods, there is a clear need to make IS easier to apply and more intuitive for diverse researchers and implementers who are not IS experts. By making IS TMFs more digestible to the broader community, there is greater potential to mend the “leaky pipeline” and increase the relevance and impact of research. Others have recently published on such tools that aim to simplify the process of applying IS TMFs and methods. These tools include a questionnaire to guide self-assessment of contextual alignment [23], questionnaires to assess context using a specific TMF [24, 25], and a stepwise process for planning clinical trials [26].

The increasing number of initiatives to simplify IS TMFs and methods is encouraging, but more work is needed to provide more comprehensive guidance across all stages of implementation and for diverse audiences in ways that allow for flexible or pragmatic application across diverse EBPs, especially if a goal is to create generalizable and sustainable programs [3, 27]. Because context changes dynamically, such efforts should support iterative consideration of contextual alignment longitudinally across the lifecycle of a program’s planning, implementation, and sustainment stages [28, 29].

We addressed these issues by creating an interactive webtool for diverse audiences to iteratively apply an IS TMF, the Practical, Robust Implementation and Sustainability Model (PRISM) [9]. PRISM was selected for multiple reasons. First, PRISM is intended to be used pragmatically and iteratively across the lifecycle of an EBP to maximize the fit to multilevel context while considering the impact on pragmatic clinical and implementation outcomes [9, 30, 31]. Its inclusion of both contextual determinants and (RE-AIM—Reach, Effectiveness, Adoption, Implementation and Maintenance) implementation outcome measures facilitates consideration of the interrelationship between contextual alignment and outcomes [9, 30, 32, 33]. PRISM is the contextually expanded version of the RE-AIM framework, which are the outcomes included as part of the PRISM framework. RE-AIM considers key implementation and effectiveness outcomes from diverse perspectives and their representativeness. As the most widely used IS evaluation TMF [31, 32, 34], the breadth of familiarity with RE-AIM may make the contextually expanded PRISM easier to digest and understand. PRISM’s contextual domains also consider multisectoral representation to promote representativeness and equity, which are key considerations for any EBP [31, 35, 36].

We selected a webtool as the format for this work, because of its public accessibility, relative ease to sustain and modify once developed, and its automation capabilities. Through automation, a webtool can be interactive and guide users through the process of applying IS methods while also offering real-time, individualized feedback to improve their EBP’s contextual alignment.

The purpose of this article is to describe the iPRISM webtool (iterative, Practical, Robust Implementation and Sustainability Model), findings from our human-centered design process, and how this interactive tool operationalizes PRISM to guide and assess an EBP’s (a) alignment with context, (b) progress on implementation outcomes, (c) required adaptations, and (d) future sustainability and scalability across the implementation lifecycle. We provide suggestions of how the webtool can be used, discuss its strengths and limitations, and make suggestions for future research and practice. In future work, we will share the findings from ongoing evaluations of the webtool’s usability and impact across diverse settings, content areas, EBPs, and users.

## Methods

We applied a human-centered design process to co-create an interactive IS webtool to be broadly applicable across EBPs for diverse types of individual users and implementation teams. The goals of the webtool were to pragmatically guide users through the process of iteratively assessing, aligning, and adapting EBPs and implementation strategies to both current context and progress on outcomes to optimize outcomes of uptake, implementation, and sustainment.

### Development of PRISM assessment items

**Fig. 1:**
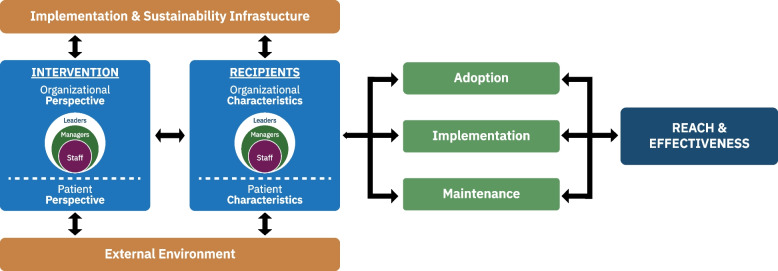
Overview of the PRISM framework which includes the context domains and RE-AIM outcome dimensions. Adapted from Feldstein and Glasgow [9]. On the left, the figure describes how PRISM is used to assess and align an intervention or program’s characteristics with the characteristics and perspectives of multiple levels of partners including patients or community members and the organizational personnel (e.g., leadership, managers, staff) as well as the implementation and sustainability infrastructure (e.g., resources) and external environment (e.g., policy, guidelines). The figure also demonstrates how contextual alignment of an intervention influences the impact or outcomes. On the right of the figure are PRISM’s RE-AIM outcomes of Reach, Effectiveness, Adoption, Implementation, and Maintenance, which have interdependencies

As described above, the PRISM framework, which includes the pragmatic RE-AIM outcomes was selected to address these goals. Figure 1 provides an overview of PRISM which consists of multilevel PRISM context domains and the RE-AIM outcomes. The 6 context domains include (1) organizational or setting characteristics, (2) patient or community characteristics, (3) patient or community perspectives on the EBP, (4) organizational perspectives on the EBP, (5) the implementation and sustainability infrastructure, and (6) the external environment. The RE-AIM outcomes include the five dimensions of reach, effectiveness, adoption, implementation, and maintenance. To provide users direction on how to operationalize PRISM context domains and RE-AIM dimensions, a set of prompting assessment questions were developed. These assessment items were initially developed by the research team, informed by our prior experience applying PRISM and RE-AIM in diverse contexts [37–39], and then iteratively refined throughout the human-centered design process. The purpose of these assessment items is to capture the general perceptions of individual users regarding potential areas for improvement and to facilitate discussion among teams. The first set of assessment items was drafted by two members of the research team (RG and BR). These items were created to align with the PRISM context domains and RE-AIM outcomes and were formulated to be broad in terms of applicability to the types of contexts, populations, and interventions. Members of the national RE-AIM Working Group [40] provided the first round of feedback for the refinement of the items. Most changes in this stage were related to the wording of the items for clarity and the most appropriate anchors for the response scale. During the human-centered design process, feedback from the participants on the wording and response option for the items were documented. Differing perceptions across team members are anticipated and valued and there are no “right answers or ultimate criterion” against which to validate responses; thus, interrater reliability testing was not relevant.

### Human-centered design process

We convened a trans-disciplinary research team to guide the design process, which included English and Spanish-speakers with expertise in IS, clinical informatics, behavioral science, human factors engineering, computer science, public health, global health, health equity, pharmacy, and health care. The research team engaged multisectoral individuals and teams of researchers and practitioners who would be likely users of the webtool. Researchers and practitioners were identified using convenience and snowball sampling and included a wide range of perspectives and types of EBPs, including government, public health, chronic and acute health care, and community settings as well as different levels of IS expertise (none to expert).

The human-centered co-creation process consisted of two consecutive, iterative phases that led to the current version of the webtool: (1) design and (2) usability testing. Each of these phases included use of progressively higher fidelity prototypes. The various prototypes allowed us to nimbly refine the webtool within our resource constraints, notably given the expense of web developer time. In each of these phases, users were asked to follow the “think aloud” method [41] and simulate or apply the webtool to an EBP they were familiar with. The think aloud protocol involves participants verbalizing their actions and thoughts throughout to gain insight into their thought processes [41].

The simulations represented EBPs at various stages, including pre-implementation planning, implementation, and sustainment. At the end of each simulation, users were asked semi-structured questions about the experience and how they might use the actual webtool. Types of questions asked to understand usability assessed likelihood of using or recommending the webtool to others, whether more direction/instruction was needed to use the webtool, and if there were confusing aspects of the webtool. Between each simulation, the research team discussed the findings and made changes to the prototype as appropriate. Decisions of whether to make changes balanced user requests with resource availability and evidence-based principles of human-computer interaction, which considers the impact of usability errors on user experience and usefulness of a technology [42].

Design testing entailed the research team developing initial, low-fidelity Excel-based prototypes of the webtool. The Excel-based prototypes consisted mostly of static images and allowed for minimal interactivity. The focus of design testing was to validate the general direction of the planned user experience and refine the content, including wording of the assessment questions and the types of graphical feedback displays. Because of the low fidelity of the prototype at this phase, participants had limited ability to simulate use of the webtool, but they were asked to think aloud as they reviewed the prototype, followed by a semi-structured discussion.

Usability testing included higher-fidelity Adobe XD prototypes of the webtool that allowed for more interactivity and functionality (e.g., hover states, clickable links, toggling between pages). The higher fidelity prototype more closely resembled what the actual webtool might look like and allowed users to simulate how they would apply the webtool to an actual EBP with minimal input required from the research team. The purpose of usability testing was to identify any ergonomic issues and optimize ease of use, which included considerations of flow and identification of usability errors. Usability errors are defined as characteristics that cause confusion or limit its potential to assist users in applying PRISM. At this stage of testing with prototypes, usability was evaluated qualitatively and did not include validated quantitative assessments.

Based on input from design and usability testing, an external web developer (Insight Designs LLC; Boulder, CO) built the current version of the webtool. The webtool will undergo additional usability and user testing, including validated assessments to quantify usability, acceptability, and feasibility of the actual webtool, which will be described in detail in a future paper. In the spirit of rapid dissemination and agile design, the current version of the webtool is publicly available for use and is described in detail here.

## Results

Based on feedback from 28 potential target users, including those with and without IS or PRISM expertise, we refined the webtool’s purpose, content, navigation, target audience, and visual displays to optimize the user experience. Table 1 summarizes the type of feedback and usability errors identified during the think aloud and semi-structured discussion as well as the rationale for whether changes were made based on the research team’s discussion. When asked about likelihood of using or recommending the webtool to others, users generally expressed interest in using the webtool and high likelihood of recommending it to others. During the simulated usability testing with a high-fidelity prototype, users were generally able to complete the webtool without additional support or clarification.

**Table 1:** Description of feedback or usability errors from user-centered design process and decisions made

**Description of feedback or issue *(selective quotes)***	**Action taken with description or rationale for no action taken**
Add more description to differentiate the stages or phases of implementation	Action taken Clarified wording and added info button hover for more explanation.
Add an option to select more than one stage or phase of implementation	No action taken The research team was unable to think of an example when this is needed as user can only work on one stage at a time and can return later for other phases. Only 1 person mentioned this.
Provide more clarity upfront on what the purpose of the tool is, what to expect when completing the tool, an estimate of how long it takes to complete, how data/responses will be used, and what the end result or product will be. *(“What happens when I exit, do I return to the Home page?”)*	Action taken Added more details, including clear outline of steps involved in completing the tool and a time estimate as well as reassurance that a user can save and continue later, generate reports of their answers and that their responses will only be used for internal improvement purposes.
Provide input on progress *(“I personally would like to see breadcrumbs or a road map of sorts that shows you where you are in the tool…”)*	Action taken Added progress bar.
Need to minimize time spent answering general and reflection questions	Action taken Revised language and used font formatting to emphasize goal is to “briefly” answer or consider questions and reduced the number of reflection and project description questions.
Acknowledge that a program is a broad term that might include any of Brown et al.’s 7Ps (e.g., pill, product, policy)	Action taken Added this to the general questions page when asking a user to reflect on their program.
Explain what PRISM and RE-AIM are at the very beginning. *(“It was assumed that the user was already familiar”)*	Action taken Replaced an existing “Learn More About PRISM” from the body of the homepage with an “About PRISM” button in the top right of all web pages. Provided more details within “About PRISM” regarding what PRISM and RE-AIM are along with additional references.
Request for additional videos, including examples of using the webtool and how to interpret the summary results report *(“…the intro video was great. I might add a second video to walk through practical application.”)*	No action taken Will continue to assess if others also recommend this. In future, will add additional videos including examples of how to use the webtool.
Be consistent with word choice (e.g., website vs webtool)	Action taken Changed to webtool throughout.
Improve visualization, including general esthetics and use of font formatting for emphasis throughout. *(“…home page could use a bit of color.”)*	Action taken Iteratively made improvements to overall visual look of the webtool.
Provide more direction on how a user should answer the assessment questions during the planning, pre-implementation phase *(“I worry that some individuals will use aspirational ratings and thus not identify areas of weakness.”* *“I think that it might be difficult for folks, to estimate the likeliness of the RE-AIM elements.”)*	Action taken Added instructions to provide “…your best estimate based on your knowledge at the time of completion. There are no ‘right’ answers. These questions are meant to be thought-generating.”
Have each of the assessment items for each RE-AIM and PRISM construct on separate pages *(“It seems daunting to scroll through all on one screen”)*	No action taken There is a value to have users on the same page and allow for scrolling up and down as they complete these. Only one user mentioned this.
Preferred format for response items was generally slider scale bar, but some preferred radial dials	Action taken Changed original ‘drop downs’ to slider scale bars
For the slider bar, allow for selecting a non-whole number (e.g., 2.5)	No action taken This is an ideal state, but the cost of implementing the change was prohibitive.
Add a not applicable option for the assessment items *(“I think it would be helpful to have a ‘Not Applicable’ option as not every component of PRISM/RE-AIM may be necessary for each project.”)*	Action taken Added “N/A” option.
Reword and reframe assessment items to align with intent, correct errors, and for clarity and ease of interpretation. *(“Make ‘perspective’ plural”* *“I’m not sure how “recipients” are different from patients or community members. In my mind -those are the same groups of people. Does recipients mean program staff?”* *“I didn’t initially understand that there were two separate questions about importance vs. effectiveness in the PRISM questions- maybe some subheaders or bolding etc. would help?”)*	Action taken Corrected typos, extra spaces, grammatical errors. Rephrased some questions including word choice and prompts. Removed separate importance and effectiveness prompts for each assessment item. Added examples and info button hovers to provide additional clarity about the intent of assessment items.
Re-order RE-AIM questions to align with the acronym	No action taken The questions are organized to align with the order in which the sequential order in RE-AIM outcomes occur, which are different than the order they appear in the acronym.
Revise radar bar graph figures to make scales and interpretation more clear *(“I like the visuals but think that there need to be a legend of what the different levels in the circle mean.”)*	Action taken Added additional explanation of how to interpret and formatting to make salient points more clear. Added numbered lines/rings to the figure.
Add more direction and examples on how to identify and select strategies *(“Consider adding a menu of options…It could be helpful to anchor the strategies based on the domains that could be improved.”* *“The strategies section is simultaneously the most important part of the website (this is what might lead to actionable steps) and the most challenging. I think you will lose many visitors here, it is very daunting to come up with strategies without more guidance on what those strategies might look like.”)*	Action taken Added example strategies that could address each construct of PRISM or RE-AIM and additional resources. The list was generated with input from the national RE-AIM Working Group. Added instructions on how to select the highest priority strategies based on impact and feasibility ratings.

### Description of the webtool

The iPRISM webtool is publicly available and can be found at https://prismtool.org. After a brief video introduction and summary of PRISM, the webtool guides users through the process of aligning an EBP with context to maximize impact during the planning, implementation, and sustainment stages of a program. It is available in English and Spanish and can be used by individuals or teams for diverse public health and healthcare related EBPs. The same PRISM context and RE-AIM outcome assessment items are used across planning, implementation, and sustainment stages, with minor modifications to the wording across stages. Users are encouraged to use the webtool early in the planning stage and repeatedly during implementation and sustainment in the spirit of designing for dissemination, equity, and sustainment [43]. The webtool can also be used separately for any stage (i.e., planning, implementation, sustainment). Before using the webtool, users should already have an idea of the context, intervention, and intended outcomes of interest.

To support users with varying degrees of IS experience while also preserving streamlined user interfaces, the webtool includes embedded education and training, including a video tutorial and use of optional links and info buttons with hover effects for additional information and examples (e.g., more information on PRISM or RE-AIM). Upon completing the webtool, users are provided tabular and graphical summaries of their assessments of fit to context and impact on outcomes as well as a prioritized list of feasible and impactful implementation strategies and formal action plans for accountability. A menu bar allows users to efficiently toggle back and forth across these components and review or modify their content or their responses.

The webtool is organized into four sequential steps, which are described in greater detail in Table 2: Step 1: Set up; Step 2: Assessment of context and impact on outcomes; Step 3: Review of assessment results; and Step 4: Identification and prioritization of implementation strategies and action planning. Whether users are completing the webtool as individuals or teams, they will complete each of these four steps, which span multiple web pages. However, for teams, there is an additional, Step 5: Team results report. The steps are described below and in more detail in Table 2.

**Table 2:** Description of the iPRISM Webtool steps

Step	Description of key content and functionality	Additional information
1—Set up	• Orients user to purpose and what PRISM is ◦ Video ◦ Multiple links to supplementary information • Allows user to select Spanish • Provides information about what to expect ◦ Can save progress and return ◦ Results can be printed ◦ Data is stored in aggregate and will not be shared ◦ Data is compliant with international data security standards, including European GDPR’s opt-in cookie privacy policy • Asks user to indicate whether they are completing as an individual or part of a team ◦ Teams are provided a unique code for individuals of a team to use, which will link their data • Asks user to indicate their EBP's stage (planning, implementation, sustainment) ◦ Responses dictate which set of assessment questions are displayed • Prompts user to enter a name for their EBP ◦ The name dynamically appears throughout the rest of the webtool • Asks general questions about the EBP to: ◦ Allow the development team to track the breadth and representativeness of the webtool’s use and make improvements ◦ Get the user thinking about the EBP goals, setting, intended recipients, what the key components are and how the components are or will be implemented	• The general questions are intended to be brief and were designed to make them more efficient (e.g., categorical selections, wording such as “briefly” or “list”)
Step 2—Assessment of context and impact on outcomes	• Guides user to systematically consider the multilevel context based on PRISM context domains and evaluate the perceived or actual impact based on RE-AIM outcome measures ◦ 6 questions to operationalize PRISM ◦ 15 questions to operationalize RE-AIM	• Questions consider representativeness/equity of impact and representation of perspectives • Questions are evaluated on a 6-point Likert scale with a slider bar and option to indicate “not applicable” • Slider bars are discreet, not continuous to align with how the results and graphical displays are reported • Encourages users to make their best estimates based on what they know and anticipate
Step 3—Review of assessment results	• Displays responses to questions in graphical figures with option to hover for more details ◦ Primary display is a radar bar chart ◦ PRISM and RE-AIM responses are displayed separately, side by side ◦ Option to hover over for additional details including specific wording of the question ◦ Option to view in table or bar graph format • Explains how to interpret the figures ◦ When the radar bar chart is more filled in (more color), that indicates greater likely success of their EBP • Prompts user to review the figures and reflect on areas with lower scores and consider general issues and areas in need of improvement • Provides space for the user to document their reflections	• PRISM responses are presented individually because there is only 1 question per domain • RE-AIM responses are presented as the mean dimension scores, because there are multiple questions per dimension ◦ User can hover over the radar bar chart to see individual responses/scores
Step 4—Identification and prioritization of implementation strategies and action planning	• Directs user to *identify strategies* to address areas that need improvement ◦ Includes link to view any reflection notes from Step 3 ◦ Includes examples of implementation strategies, organized by PRISM domains and RE-AIM dimensions that the strategies might target and have been successful in prior research ◦ Users are encouraged to consider feasibility, impact (both effectiveness and equity), and sustainability ◦ Users are asked to provide a name for each strategy (50-character limit) and offered space to take notes on each • Prompts user to *rate strategies* identified based on feasibility and impact using slider bars ◦ Slider bars auto-populated with strategy names entered ◦ Impact considers equity/representativeness of outcomes • Assists user to *prioritize strategies* based on feasibility and impact ratings using a scatterplot ◦ Feasibility and impact ratings for each strategy dynamically auto-populate a scatterplot ◦ Encourages user to prioritize strategies in upper right quadrant (highest impact and feasibility) ◦ Can be exported/printed • Offers action plan template • Reminds user to iteratively use the webtool and to assess the impact of strategies implemented	• Split into 4 collapsible sub-sections on one page ◦ Section 1 displays the PRISM and RE-AIM bar charts ◦ Sections 2 (*identify*), 3 (*rate*), and 4 (*prioritize*) dynamically auto-populate as the user proceeds • Includes a limited list of example implementation strategies that pair with the different constructs of RE-AIM and PRISM • Before ending a session, the webtool encourages users to export/print any of the graphical displays they want to save • Once the session ends, a user: ◦ Cannot return to review/edit responses ◦ Will be provided the option to download their tabular results in Excel format and have their results emailed to them • Users who use the webtool iteratively will create a new session each time
Step 5 (only for teams)—Team results report	• Provides option to view summary report of team’s responses ◦ Accessible with their unique team URL that can be bookmarked and revisited on demand and in the future, encouraging iteration ◦ Includes anonymous individual responses and the entire teams’ results summarized by mean and distribution frequency of responses for each of the assessment items	• Mean and distribution of responses allows for easy identification of areas of congruence and discrepancy in the team’s perspective • Report can facilitate team-based discussion on what areas are most important to improve, which implementation strategies to prioritize, and to create action plans

*EBP* Evidence-based practice, *GDPR* Genera, Data Protection Regulation, *PRISM* Practical, Robust Implementation and Sustainability Infrastructure, *RE-AIM* Reach, Effectiveness, Adoption, Implementation, Maintenance

#### Step 1: Set up

This step orients the user to the webtool and asks the user a number of questions about their EBP (e.g., name, setting), how they are completing the tool (individual versus team; stage of their EBP), and allows them to select the Spanish version. Responses to many of these questions dictate how the data entered will be stored and ultimately presented to the user.

#### Step 2: Assessment of context and impact on outcomes

**Fig. 2:**
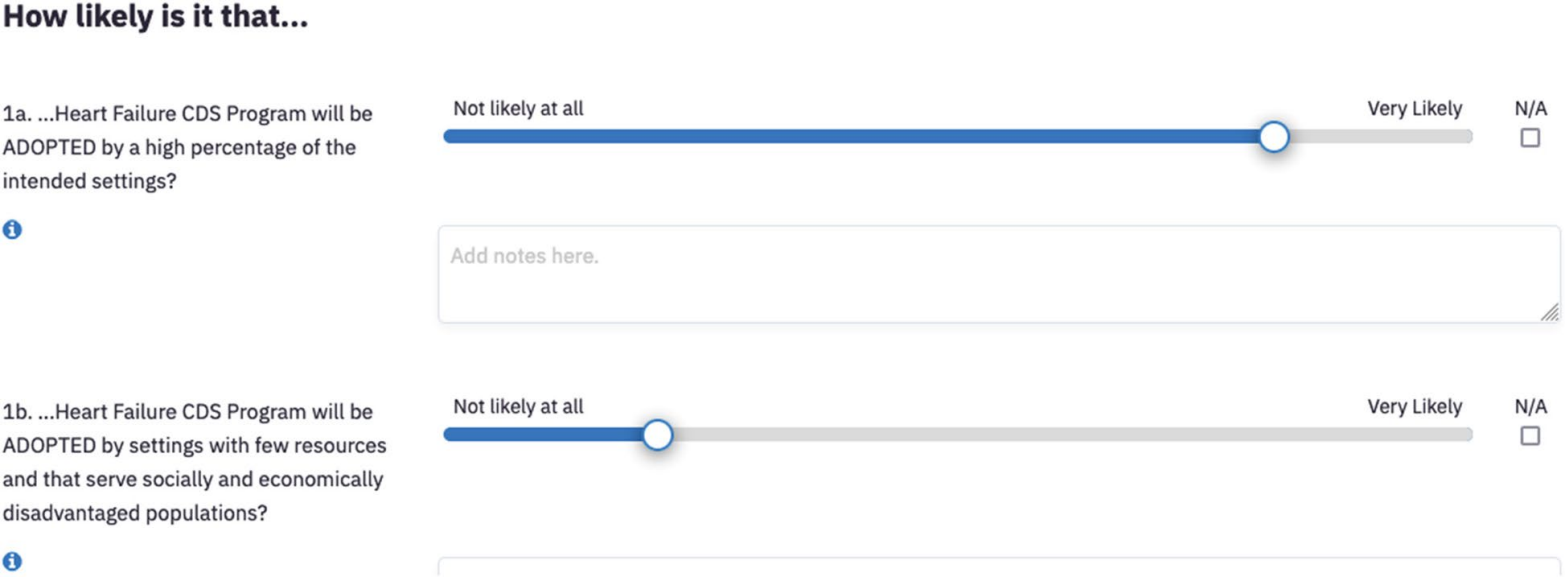
Illustration of the assessment questions and slider bars. Depicted here are two assessment questions for the RE-AIM outcome dimensions

This step guides the user to systematically consider the multilevel context (e.g., role of community members or patients and family; implementation staff; supervisors or decision makers; larger organizational setting, community and policy) based on the PRISM context domains and evaluate the estimated relationship between the contextual alignment of the EBP and the perceived or actual impact on outcomes. Impact is based on the RE-AIM outcome measures (e.g., equitable reach, implementation, sustainment). The webtool includes 21 questions to operationalize the PRISM context domains and RE-AIM outcomes, with specific consideration of representativeness and equity by asking about multi-level perspectives of the context and intervention as well as representativeness of outcomes. Figure 2 provides an illustration of the questions and slider bar response options. Additional file 1 includes the full list of the itemized questions for each stage of implementation.

#### Step 3: Review of assessment results

**Fig. 3:**
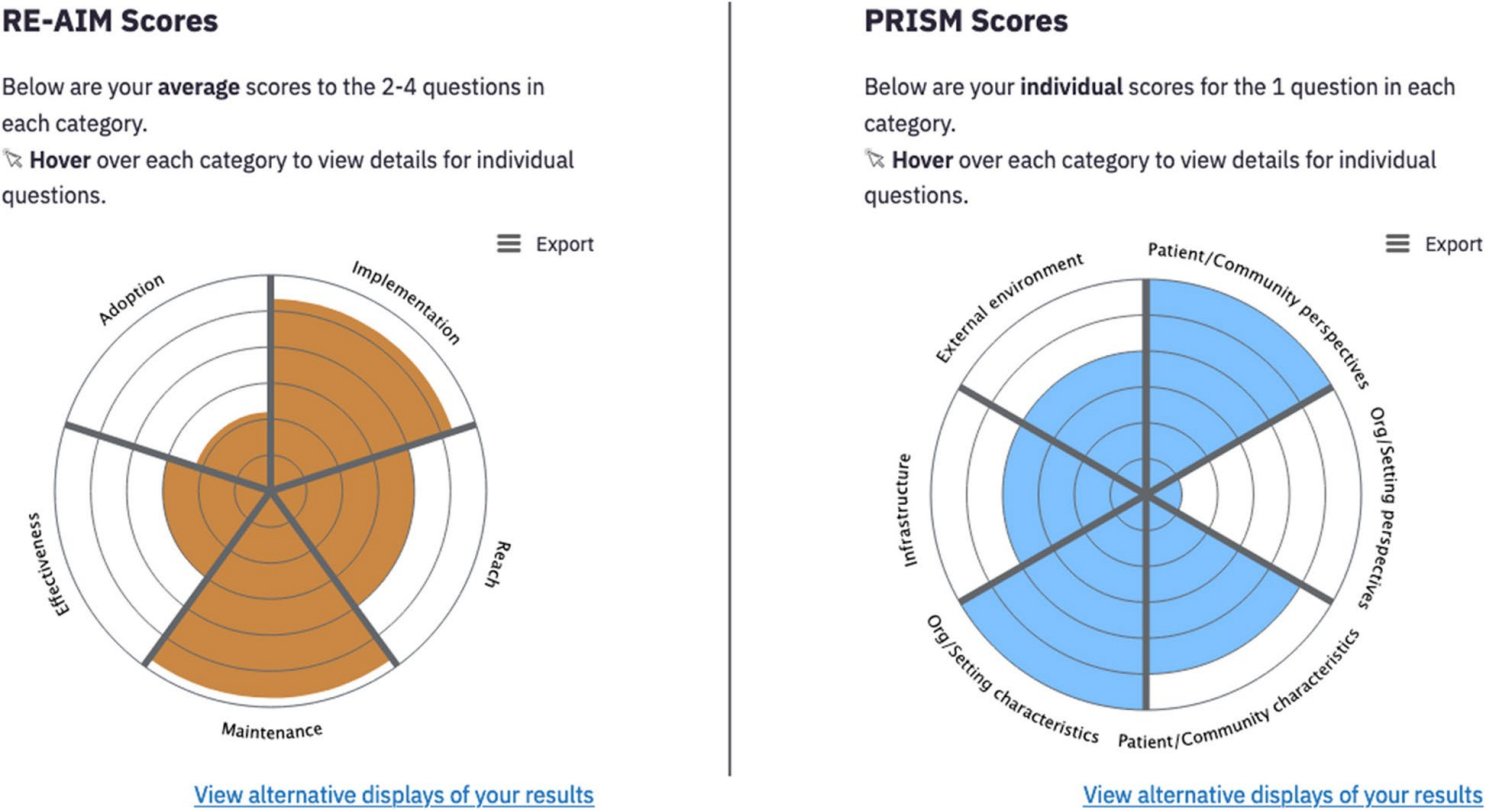
Illustration of the radar bar graphs that summarize a user’s responses. A user can opt to (1) view alternative displays (table and bar graph format), (2) export and print the figures, and (3) hover over an area of the radar bar graph to see additional details of the questions and their responses

In this step, the output or responses to the assessment questions are displayed in graphical format. Based on feedback during the design process, we selected a radar bar chart as the primary graphical display (Fig. 3) in which the PRISM and RE-AIM results are displayed side by side to facilitate consideration of the relationship between the contextual alignment of an EBP (PRISM) and outcomes (RE-AIM).

#### Step 4: Identification and prioritization of implementation strategies and action planning

In this step, the user is guided through the process of using their assessment scores to identify impactful and feasible implementation strategies that can improve contextual alignment and impact of their EBP. The definition of impactful implementation strategies includes consideration of representativeness or equity of outcomes.

The objectives of this step are slightly different depending on the stage of EBP implementation. In the planning stage, the webtool directs the user to develop strategies that optimize the initial contextual alignment of an EBP with the local setting before it is deployed with consideration of the anticipated impact on outcomes. During the implementation stage, the webtool directs the user to identify strategies for mid-course adaptations based on any changes in context and the user’s report on or perception of progress on outcomes. For the sustainment stage, the webtool directs users to identify strategies that could improve ongoing maintenance and sustainability of the EBP based on current and anticipated progress on desired outcomes.

**Fig. 4:**
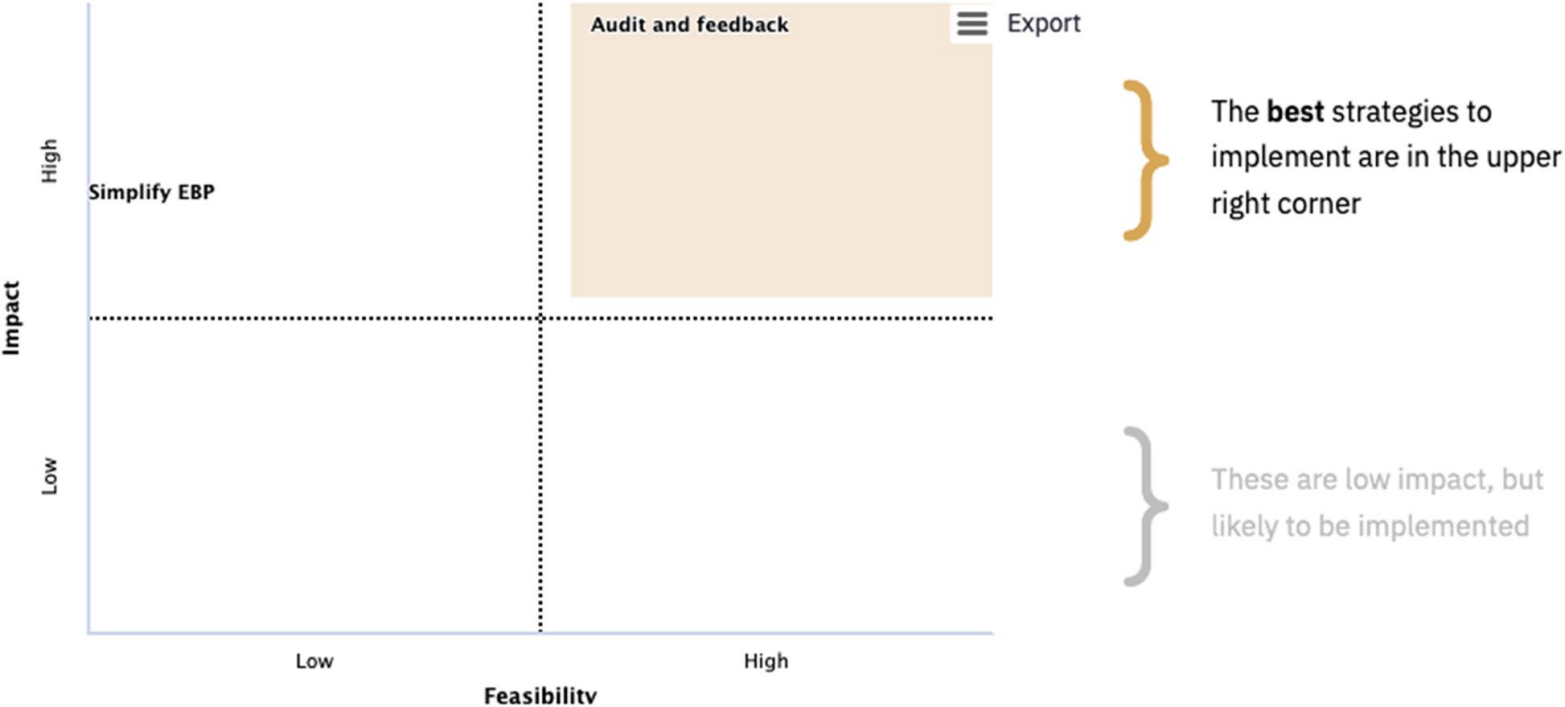
Illustration of the scatterplot that assists a user in prioritizing strategies based on impact and feasibility ratings

This step is split into 4 sub-sections on one page in which responses to one section dynamically auto-populates subsequent sections in order to guide users through the process of identifying, rating and prioritizing strategies or adaptations. As illustrated in Fig. 4, the webtool prompts users to prioritize those strategies with the highest feasibility and impact ratings, which are displayed in the upper right quadrant of a scatterplot. After reviewing the scatterplot, users are encouraged to consider adjusting their implementation strategies if the impact and feasibility ratings are suboptimal. Changes to the implementation strategies will dynamically update the subsequent sections. At the end of this component, users are offered a template to create a formal action plan for the implementation strategies they prioritized based on feasibility and impact. If users are done, they can then select a button to complete their assessment, which will end their session.

#### Step 5 (only for teams): Team results report

**Fig. 5:**
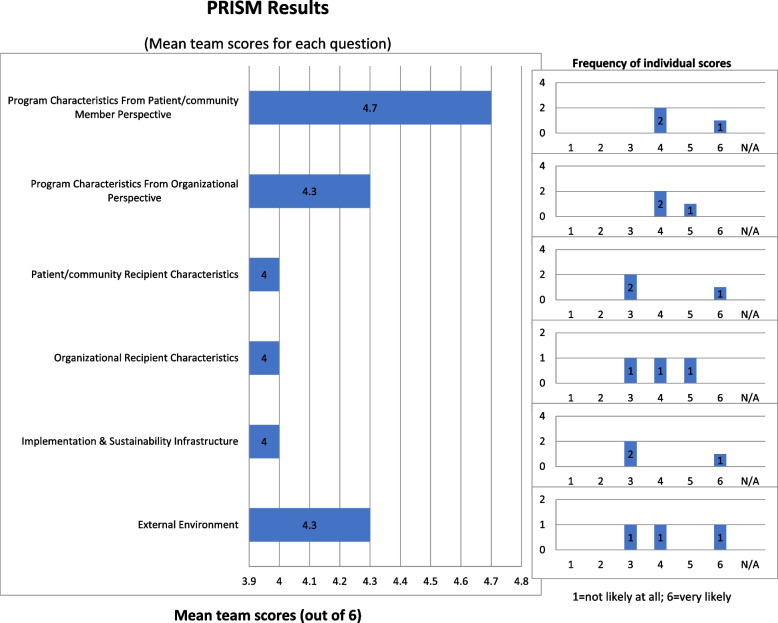
Illustration of the team summary report. Depicted here is the PRISM team summary report. The RE-AIM results are also summarized similarly for teams

Users who complete the webtool as part of a team will have the option of viewing and exporting their team’s responses. Figure 5 includes an example of what the team report includes.

## Discussion

The iPRISM webtool advances IS by making its TMFs and approaches more user-friendly for the broad community of researchers and implementers with and without IS expertise. The webtool aims to simplify the theoretical PRISM context domains and RE-AIM outcomes by using actionable assessment questions and then guiding users through the process of identifying and prioritizing strategies to align and adapt an EBP with the context (and their progress when used in later stages). By focusing on this broad audience, the webtool has potential to result in greater adoption of IS TMFs and methods. For experienced implementation scientists, the iPRISM webtool may be used to support grant submissions or as a resource when collaborating with implementation teams with minimal to no IS experience.

The interactive feedback and guidance provided by the iPRISM webtool is similar in nature to the Program and Clinical Sustainability Assessment Tools (PSAT/CSAT) [44, 45]. While the PSAT/CSAT focuses on assessing the sustainability of an EBP [44, 45], our webtool focuses on how EBP contextual alignment can be leveraged to optimize not just sustainability but also other implementation and effectiveness outcomes (e.g., reach, adoption, effectiveness). Our webtool is not the first tool to guide assessment, alignment, and adaptation of an EBP to the implementation context [23–26, 46, 47], but it is to our knowledge the first web-based interactive resource. In addition, and in contrast to other available tools, it integrates context with outcomes and is fully automated. The interactive design of our webtool aims to improve ease of use by guiding users through the process with prompts and makes the process more efficient by summarizing assessments with individualized feedback on how to improve contextual alignment and outcomes. Other strengths of our webtool are the guidance to identify, evaluate, and prioritize impactful implementation strategies as well as the prompts to create formal action plans for execution and accountability.

Although the web-based format of our tool is a strength, it does present some notable challenges. First, the cost of contracting with a web developer to build the webtool was relatively high, totaling an estimated $100,000. Table 3 illustrates the varying costs of different features of the webtool and translation. It was also difficult to identify a compatible web developer that was both within our budget and that possessed the skillset we desired. We sought a web developer that not only had a track record of developing well-designed websites, but that was also able to provide expertise related to best practices in web design, including data security standards, accessibility, and human-computer interaction. Ultimately, we found a web developer that met our needs and augmented their skill by embedding within our research team an academic-based expert in human-computer interaction, data visualization, and software design (author SD). While the initial cost of developing the webtool was high, ongoing maintenance is anticipated to be low, limited to website domain and hosting costs and the research team’s in-kind time. To facilitate sustainability, the web developers strategically built the website to grant the research team administrative access and ability to make changes over time, thus averting the need for ongoing maintenance costs from the web developer.

**Table 3:** Description of web development costs for different features of the webtool

Select features and tasks completed by the web developer	Cost
Install and configure WordPress	$1014
Connect Spanish translation plugin	$8450
Customize theme	$4056
Create custom assessment question forms	$8112
Create custom primary graphical results displays	$5408
Create custom alternative graphical results displays	$8112
Dynamic form additions/customizations for strategies page	$7436
Create unique URL method for data re-access	$1352
Create custom Excel results export	$4056
Functionality to facilitate team completion and summary reports	$18,252
Create method to email results to users	$14,534
Iterative pre-launch testing	$4056
Training iPRISM team to have administrative access	$507
Site launch	$338
Project management	$9802
Privacy compliance	$5408
Translate English to Spanish^a^	$1397
**Total cost**	**$102,290**

These cost estimates do not include the time required from the research team or user testing

*iPRISM* iterative Practical, Robust Implementation and Sustainability Infrastructure

^a^The translation was completed by a third-party translation service, not the web developer

## Limitations

There are also limitations to the current webtool. Although we aimed to create an intuitive tool that could be used across multiple audiences with varying degrees of IS expertise, the webtool would still benefit from further refinements to minimize jargon and to expand its relevance to the global community of researchers and implementers, including different Spanish-speaking contexts and other languages. We tested and refined the webtool with Spanish speakers, with an emphasis on select countries in Latin America, potentially limiting its cultural relevance to other Spanish speaking settings. It is also unclear how much facilitation from someone with IS expertise different types of webtool users require or if users with limited IS experience have enough guidance from the webtool alone to select implementation strategies. The webtool described here is also not intended to be the final product but rather a living tool that will be refined over time. As of yet, we have not yet comprehensively tested the usability or acceptability of the webtool. However, we have formatively evaluated usability and acceptability with prototypes of the webtool and found them to be satisfactory based on user report of interest using the webtool and recommending it as well as ability to use the prototypes without needing additional direction/instruction; thus, we deemed the webtool ready for dissemination. Finally, due to web development cost and time constraints, we were not able to develop all design features that we and our implementation partners desired, such as a professionally formatted PDF report that included all figures and tabular results or the ability to save and return to a given assessment when using the webtool iteratively.

In future work, we will continue to refine the webtool to make it intuitive for diverse audiences in different settings. We are actively continuing user testing of the webtool and will continually update the webtool based on this work. As we are able, we will continue to add advanced design features, including features that will allow the user to customize the tool based on their preferences. We will also prioritize adding design features and automation that make the user experience more intuitive and efficient, in addition to continually de-jargonizing the language and content. To appeal to the informational needs of our broad intended audience, we will also continue to embed additional, optional training and tutorials, including examples of how the webtool can be used. Other areas for future work include testing the webtool under different conditions (e.g., with or without a facilitator for individuals or teams), assessing how and when the tool is used over time, and evaluating the impact of using the tool on various outcomes (e.g., user perceptions, project specific primary aims, RE-AIM outcomes, social and equity impacts).

## Conclusion

In summary, we have created a new webtool designed to make it easy for diverse researchers and implementers to assess, align, and adapt EBPs to a specific clinical or public health issue using PRISM. By simplifying the use of PRISM and making it more actionable, this webtool is anticipated to increase uptake of this IS framework by more diverse audiences of researchers and practitioners, thereby resulting in more research that is relevant, reproducible, and sustainable. As IS advances, there is a clear need for ongoing development of this webtool and similar resources to make TMFs more intuitive and approachable. Future work should prioritize development and evaluation of user-friendly approaches to apply other TMFs and to guide sustainment and adaptations of EBPs.

### Supplementary Information

**Additional file 1.** iPRISM Webtool Assessment Questions.

## Data Availability

https://prismtool.org/

## Abbreviations

IS: Implementation science
iPRISM: Iterative Practical, Robust Implementation and Sustainability Model
PRISM: Practical, Robust Implementation and Sustainability Model
TMFs: Theory, models, and frameworks
EBP: Evidenced-based programs
RE-AIM: Reach, Effectiveness, Adoption, Implementation and Maintenance
PSAT/CSAT: Program and Clinical Sustainability Assessment Tools
