# Supplementary material for: The iPRISM webtool: an interactive tool to pragmatically guide the iterative use of the Practical, Robust Implementation and Sustainability Model in public health and clinical settings
Source: Implement Sci Commun. 2023 Sep 19;4:116. doi: 10.1186/s43058-023-00494-4 (PMC10508024; doi:10.1186/s43058-023-00494-4)
Supplement: Supplementary file 1 — Additional file 1. iPRISM Webtool Assessment Questions. [file 43058_2023_494_MOESM1_ESM.docx]

| **Additional file 1. iPRISM Webtool Assessment Questions** | |
| --- | --- |
| **RE-AIM Outcome Dimension Assessment Items** | |
|  | *All response options used a 6-point Likert scale and varied by the stage of implementation as described below:*  Pre-implementation planning stage (Pre-imp): Not at all likely - Very likely  Implementation stage (Imp):* Not at all - To a great extent  Sustainment stage (Sust): Not at all likely - Very likely  **For the implementation stage, question 5a of the maintenance area uses options of Not at all likely – Very likely* |
|  | **Area 1: Adoption** |
|  | ***1a.*** *Adoption Setting Level: The number and percent of those settings (e.g., clinics, worksites, schools) invited who agree to participate in a program.*  Pre-imp: How likely is it that your program will be adopted by a high percentage of the intended settings?  Imp: To what extent is your program being adopted by a high percentage of the intended settings?  Sust: How likely is it that your program will be adopted by a high percentage of **new** settings? |
|  | ***1b.*** *Setting Adoption Representativeness: Considers if those settings with the fewest resources and serving socially and economically disadvantaged clientele are as likely to participate as other settings.*  Pre-imp: How likely is it that your program will be adopted by settings with few resources and that serve socially and economically disadvantaged populations?  Imp: To what extent is it your program being adopted by settings with few resources and that serve socially and economically disadvantaged populations?  Sust: How likely is it that your program will be adopted by new settings with few resources and that serve socially and economically disadvantaged populations? |

|  | ***1c.*** *Adoption Staff Level: The number and percent of those staff (e.g., clinicians, teachers, nurses) invited who agree to participate in a program.*  Pre-imp: How likely is it that a high percentage of staff will participate in your program?  Imp: To what extent does a high percentage of staff participate in your program?  Sust: How likely is it that a high percentage of staff will participate in your program going forward? |
| --- | --- |
|  | ***1d.*** *Adoption Staff Representativeness: Considers if those staff who participate will be similar to those who decline.*  Pre-imp: How likely is it that staff who participate in your program will be similar to those who decline?  Imp: To what extent is staff who participate in your program is similar to those who decline?  Sust: How likely is it that staff who participate in your program in **new** settings will be similar to those who decline? |
|  | **Area 2: Implementation** |
|  | ***2a.*** *Implementation Staff Level: Refers to how the program is delivered in participating settings and is concerned with fidelity to core functions (or components), adaptations to the form of the program, and the costs and resources required.*  Pre-imp: How likely is it that staff will consistently deliver the core functions (or components) of your program with high quality?  Imp: To what extent is the staff consistently delivering the core functions (or components) of your program with high quality?  Sust: How likely is it that staff will continue to deliver the core functions (or components) of your program with high quality? |
|  | ***2b.*** *Implementation Setting Level: Refers to how the program is adapted by the setting.*  Pre-imp: How likely is it that your program will be adapted as needed to fit your setting?  Imp: To what extent is your program is being adapted as needed to fit your setting?  Sust: How likely is it that your program will be adapted as needed to fit your setting in the future? |

|  | ***2c.*** *Implementation Infrastructure: Refers to the costs and resources required.*  Pre-imp: How likely is it that the costs and resources needed to deliver the program are feasible for your setting?  Imp: To what extent the costs and resources needed to deliver the program are feasible for your setting?  Sust: How likely is it that the costs and resources needed to deliver the program will be feasible for your setting in the future? |
| --- | --- |
|  |  |
|  | **Area 3: Reach** |
|  | ***3a.*** *Reach Individual Level: Number and percent of those who participate of those who are invited or eligible (i.e., intended recipients/beneficiaries).*  Pre-imp: How likely is it that your program will reach a high percentage of its intended recipients (e.g. patients, employees, students)?  Imp: To what extent is your program reaching a high percentage of its intended recipients (e.g. patients, employees, students)?  Sust: How likely is it that your program will reach a high percentage of its intended recipients (e.g. patients, employees, students) in the future? |
|  | ***3b.*** *Reach Representativeness: Who is intended to benefit and who actually participates, including the extent to which there are equity concerns related to participation.*  Pre-imp: How likely is it that your program will equitably Reach the intended recipients, including populations that are socially and economically disadvantaged?  Imp: To what extent is your program equitably reaching the intended recipients, including populations that are socially and economically disadvantaged?  Sust: How likely is it that your program will equitably reach the intended recipients (e.g., patients, workers, students, community members), including populations that are socially and economically disadvantaged in the future? |

|  | **Area 4: Effectiveness** |
| --- | --- |
|  | ***4a.*** *Effectiveness Individual Level: Whether your program is achieving its goals and its impact on your key outcomes. Effectiveness also includes the program’s impact on quality of life and any negative effects.*  Pre-imp: How likely is it that your program will be effective?  Imp: To what extent is your program effective?  Sust: How likely is it that your program will be effective in the future? |
|  | ***4b.*** *Effectiveness Representativeness: The variability in outcomes across participants, including the extent to which there are equity concerns.*  Pre-Imp: How likely is it that your program will be effective for intended recipients that are socially and economically disadvantaged?  Imp: To what extent is your program effective for intended recipients that are socially and economically disadvantaged?  Sust: How likely is it that your program will be effective for intended recipients that are socially and economically disadvantaged in the future? |
|  | **Area 5: Maintenance** |
|  | ***5a.*** *Maintenance Setting Level: The extent to which a program continues to be delivered (with appropriate adaptations as needed) to become part of the routine organizational practices, at a minimum follow-up of one year and preferably two or more years.*   Pre-imp: How likely is it that your program will continue to be delivered over time in a high percentage of participating settings?  Imp: How likely it is that your program will continue to be delivered over time in a high percentage of participating settings?  Sust: How likely is it that your program will continue to be offered over time in a high percentage of participating settings in the future? |

|  | ***5b.*** *Maintenance Setting Level: The extent to which a program continues to be delivered (with appropriate adaptations as needed) to become part of the routine organizational practices, at a minimum follow-up of one year and preferably two or more years.*  Pre-imp: How likely is it that your program can be adapted as needed so that it continues to produce high quality results?  Imp: To what extent your program will continue to be adapted as needed so that it continues to produce high quality results?  Sust: How likely is it that your program can be adapted as needed so that it continues to produce high quality results in the future? |
| --- | --- |
|  | ***5c.*** *Maintenance Individual Level: The extent to which the program effectiveness is sustained over time.*  Pre-imp: How likely is it that will your program show **sustained** effectiveness (at minimum 1-2 years)?  Imp: How likely is it that will your program show **sustained** effectiveness (at minimum 1-2 years)?  Sust: How likely is it that your program will show **sustained** effectiveness (at minimum 1-2 years)? |
|  | ***5d.*** *Maintenance Individual Level: The extent to which the program effectiveness is sustained over time.*  Pre-imp: How likely is it that your program will show sustained effectiveness over time (at a minimum 1-2 years) for socially and economically disadvantaged populations?  Imp: How likely is it that your program will show sustained effectiveness over time (at a minimum 1-2 years) for socially and economically disadvantaged populations  Sust: How likely is it that your program will show sustained effectiveness over time (at a minimum 1-2 years) for socially and economically disadvantaged populations? |

|  | |
| --- | --- |
| **PRISM Context Domain Assessment Items** | |
|  | *All response options used the same 6-point Likert scale of Not at all likely - Very likely.* |
|  | **Area 1: Program characteristics from the perspective of the patients or community members** |
|  | *This domain is concerned with how the people receiving the program find the program’s components to be useful or beneficial. Think about multiple types of eventual beneficiaries of the program.*  Pre-imp: How well does your program as currently planned align with the expectations/perspectives of the intended **patients or community members**?  Imp: How well does your program currently align with the expectations/perspectives of the intended **patients or community members**?  Sust: How well does your program align with the expectations/perspectives of the intended **patients or community members** to support future success? |
|  | **Area 2: Program characteristics from the perspective of the organizational (setting) stakeholders** |
|  | *This domain is concerned with how the people receiving the program find the program’s components to be useful or beneficial. Think about multiple types of* ***organizational (setting) stakeholders*** *- all members of the delivery team.* |
|  | Pre-imp: How well does your program as currently planned align with the expectations/perspectives of the **organizational (setting) stakeholders**?  Imp: How well does your program currently align with the expectations/perspectives of the **organizational (setting) stakeholders**?  Sust: How well does your program align with the expectations/perspectives of the **organizational (setting) stakeholders** to support future success? |

|  | **Area 3: Recipient characteristics - patients or community members** |
| --- | --- |
|  | *This domain is concerned with the characteristics of the patient or community member recipients of the program that is being developed, or implemented, or sustained/scaled. Think about recipients who will be eventual beneficiaries of the program.* |
|  | Pre-imp: How well does your program as currently planned align with the characteristics of the intended patients and/or community members?  Imp: How well does your program align with the characteristics of your patients and/or community members?  Sust: How well does your program align with the characteristics of your patients and/or community members to support future success? |
|  | **Area 4: Recipient characteristics - organizational (setting) stakeholders** |
|  | *This domain is concerned with the characteristics of the organizational stakeholders of the program that is being developed, or implemented, or sustained/scaled. Think about recipients who are involved with decision making or delivering the program and consider these recipients at multiple levels.* |
|  | Pre-imp: How well does your program as currently planned align with the characteristics of the organizational stakeholders?  Imp: How well does your current program align with the characteristics of the organizational stakeholders?  Sust: How well does your current program align with the characteristics of the organizational stakeholders to support future success? |
|  | **Area 5: Implementation and Sustainability Infrastructure** |
|  | *This domain is concerned with the implementation and sustainability infrastructure for the program that is being developed, or implemented, or sustained/scaled. Think about a diverse set of resources and structures that might influence the success of the initial program or continuing in the future.* |
|  | Pre-imp: How well does your program align with the available resources, staff, workflow, responsibilities and support functions to produce success?  Imp: How well does your program align with the available resources, staff, workflow, responsibilities and support functions to produce success?  Sust: How well does your program align with the available resources, staff, workflow, responsibilities and support functions to produce future success? |

|  | **Area 6: External environment** |
| --- | --- |
|  | *Think about a diverse set of resources and structures that might influence the success of the program.* |
|  | Pre-imp: How well does your program align with the characteristics of the external environment (e.g., policies, guidelines, norms)?  Imp: How well does your program align with the characteristics of the external environment (e.g., policies, guidelines, norms)?  Sust: How well does your program align with the characteristics of the external environment (e.g., policies, guidelines, norms) to support future success? |
